# Characterizing the time of day and year of falls in people with probable Parkinson’s disease

**DOI:** 10.1038/s41598-025-17752-1

**Published:** 2025-09-12

**Authors:** Daniel S. Peterson, Linda Johansson, Björn Westerlind, Thaís Lopes de Oliveira, Deborah Finkel

**Affiliations:** 1 Institute of Gerontology, School of Health and Welfare, Jönköping University, Jönköping, Sweden; 2 College of Health Solutions, Arizona State University, Phoenix, AZ USA; 3 Department of Geriatrics, Jönköping University, Region Jönköping County Jonkoping, Sweden; 4 Department of Health, Medicine and Caring Sciences, Linköping University, Linköping, Sweden; 5 Department of Geriatrics, County Hospital Ryhov, Region Jönköping County, Jonkoping, Sweden; 6 Department of Medical Epidemiology and Biostatistics, Karolinska Institutet, Stockholm, Sweden; 7 Center for Economic and Social Research, University of Southern California, Los Angeles, USA

**Keywords:** Falls, Parkinson’s disease, Time of day, Time of year, Older adults, Diseases, Health care, Medical research, Neurology, Neuroscience

## Abstract

Temporal aspects of falls are not well characterized in people with Parkinson’s disease (PD), particularly those later in life. The purpose of the current study is to characterize the timing (across day and year) of falls in people with probable PD compared to non-PD peers. Swedish registries were used to identify 441 people (mean age, 83.7 years, range = [59.0–100.0]) with one or more prospectively registered falls over the course of approximately 2.5 years. Of these participants, 40 were prescribed drugs of the class N04 “Anti-Parkinson Drugs”. Of these 40, 30 were prescribed dopamine or dopamine derivatives, suggesting presence of PD, and 10 were prescribed “other PD-related medications”. Given the unclear nature of these 10 participants, they were excluded from all analyses. Chi-squared tests and Generalized Estimating Equations were used to assess the time of day and year of falls in people with probable PD (*n* = 30) and non-PD (*n* = 401) accounting for covariates (age, gender, number of prescription medications). There was a statistically-significant effect of PD-group on fall time of day (accounting for gender, age, & number of prescription medications), such that Swedish residents with probable PD fell less often in the evening than the day (*p* = 0.015, OR = 0.61). Across the whole sample, female participants fell less frequently at night than the day (*p* = 0.032, OR = 0.73). People with probable PD fell more frequently in the spring months than other seasons (×2 = 32.1, *p* < 0.001). The finding that people with probable PD exhibit more falls during the day than night is consistent with previous work and extends knowledge to show a similar result in older, more frail people with PD. Characterizing the temporal nature of falls in people with PD can provide additional context to other fall circumstances, thus improving our ability to treat and predict falls in this group.

**Supplementary Information:**

The online version contains supplementary material available at 10.1038/s41598-025-17752-1.

## Introduction

Falls are a common and devastating occurrence in people with PD (PwPD)^1,2^, and understanding the circumstances of falls can facilitate their prediction and prevention. Previous studies have provided key insights into circumstances regarding place and cause of falls in PwPD^3–6^ (for review, see^7^). For example, falls in PwPD often occur indoors, result from intrinsic factors (i.e., related to underlying balance disorder)^1,3,4^, and are more frequent in the forward direction^1,6^.

An aspect of fall characterization in PwPD that is less understood is the time of day or time of year that falls occur. Identifying temporal aspects of falls can provide context to other fall circumstances. Three recent studies have assessed the timing of falls in PwPD. First, Gazibara completed a retrospective analysis of adults in Serbia with PD who fell in the previous 6 months^8^. Participants ranged from 22 to 83 years old, and falls were more common in the day than night. This group then completed a 1-year prospective falls assessment in 120 adults with PD in Serbia (mean age = 60.0) who denied having fallen in the previous 6 months. Again, falls were more common in the daytime than the evening^6^, and 54% of fallers were recurrent fallers^9^. Finally, Castro and colleagues tracked falls prospectively in 225 PwPD living in Brazil (mean age = 70.7 years) for 1 year^4^. One hundred and eleven (49%) fell at least once, and 76% were classified as recurrent fallers. Consistent with previous work, falls were more common in the daytime than night.

Together, these reports provide key information on circumstances of falls, and suggest daytime falling is common in PwPD. However, they have some limitations, including: 1) moderate to short follow-up period (= < 1 year), relatively young cohorts (mean ages 60–70 years across studies), 3) lack of time of year reporting, and 4) no non-PD control groups. Analyses that address these gaps will provide additional information regarding the context of falls in PwPD. Therefore, the current study’s purpose was to assess time of day and year of falls in older people with probable PD relative to non-PD peers. In addition, we present characteristics of falls across probable PD and control participants (count, recurrent vs. non-recurrent, and frequency of serious falls).

## Methods

### Participants

In Sweden, there are prospectively collected registers (Senior Alert, the Prescribed Drug Registry, the Social Care Registry, the National Patient Registry, the Cause of Death Registry, and others), providing a variety of population-based health outcomes. The goal of the current study was to utilize these registries to better understand falls in people with and without probable PD. Therefore, we consolidated data from 1) the Prescribed Drug Registry (to identify medication use, including medications indicating probable PD), 2) the national quality register called Senior Alert^10^ (see also: https://www.senioralert.se/) which, from 2015–2017, tracked falls^11^, and 3) the Cause of Death Registry (to identify people who passed away over the 2015–2017 window). To link data across these registers, a Swedish twin population (Screening Across the Lifespan of Twins; SALT^12^) was used.

Ethics approval and consent to participate: Ethical approval for the research project was provided by the Swedish Ethical Review Authority in Linköping, Sweden (dnr: 2014–2635-271; 2017–549-32; 2020–04345; prior to 2020 known as the Regional Ethical Review Board; Linköping) and followed the guidelines outlined in the Helsinki declaration (World Medical Association, 2013). Informed consent was not obtained as the study was based on pseudonymized data from a national quality registry. According to Swedish regulations, individual consent is not required for registry-based research. However, the participants have given their informed consent for their information to be collected by the Senior Alert register and used for quality improvement, research, and healthcare development. No funding was used to support this work. Clinical trial number: not applicable.

The Senior Alert registry followed a total of 2903 unique participants from 2015 to 2017. These individuals exhibited relatively high levels of frailty^13^. Within the 2015–2017 window, falls were prospectively registered by health care professionals in one of the following types of care setting: hospital, health care center, home health care center, nursing home, dementia care home, short-term nursing home, or support and service specifically for persons with diminished functioning (regulated by Act 1993:387)^11^. With each fall registration, date and time of day of the fall (categorized as morning- 6:00–9:00, forenoon- 9:00–12:00, afternoon- 12:00–18:00, evening- 18:00–22:00, and night- 22:00–6:00) was recorded. This 5-bin time of day was used for our initial analyses. In addition, and to facilitate comparisons with previous work, we also dichotomized falls into “day” (morning, forenoon, afternoon) and “night” (evening and night). The registry also documented whether a ‘consequence’ occurred as a result of the fall. This included any of the following: concussion, death, head injury, other injury, prolonged hospital or external care, soft tissue injury, vertebral damage, wounding, or fracture of any of the following: arm, foot, leg, hip or thigh. For the current analysis, a fall with any recorded consequence was flagged as a “consequential fall”.

Of the above 2903 individuals in the Senior Alert registry, 441 people had at least 1 registered fall. We chose to focus our analysis specifically on this sub-cohort, as they represented a group with confirmed falls tracking. We then searched the Prescribed Drug Registry to pull all medications prescribed to this group. Of these individuals, 40 were prescribed drugs of the class N04 “Anti-Parkinson Drugs” according to the World Health Organization (WHO) Anatomical Therapeutic Chemical (ATC) classification system (https://atcddd.fhi.no/atc_ddd_index/). Of these individuals, 30 were prescribed N04BA “Dopa and dopa derivatives”. The remaining 10 were prescribed “other PD-related medications” but not Dopa and dopa derivatives. Prescription of drugs in the N04BA class is highly suggestive of the presence of Parkinson’s disease. In contrast, monotherapy other PD-related medications such as dopamine antagonists (e.g., N04BC) may indicate restless legs syndrome. As such, to take the most conservative approach, the 30 individuals prescribed N04BA were included in the “probable PD” group. The “non-PD group” consisted of people participants not taking any “Anti-Parkinson Drugs” (class N04; *n* = 401). Therefore, the 10 individuals that were prescribed PD meds in the class N04B other than dopamine or dopamine derivatives were excluded from both groups.

Notably, co-morbidities were not able to be identified, limiting our ability to fully characterize probable PD and control groups. However, the total number of prescription medications was available. This outcome tracks closely with overall health and likelihood of falls^14^ and, as noted below, was included in statistical models to account for possible differences in overall health across groups.

### Measures

In addition to falls, the following outcomes were used in analyses: Observation period was the timeframe that falls were tracked. Although falls were tracked over a 3 year period, of the 431 individuals included in the study 165 died over that period, reducing the mean follow-up time to < 3 years in each group. Age was identified at the start of the observation period. Fall counts were the number of registered falls over the observation period. Recurrent faller status was defined as > 1 falls over the observation period. “Number of medications” was defined as the number of unique medications prescribed over the observation period.

### Analyses

*Fall rates*: Group characteristics, including the counts of falls and consequential falls across probable PD and non-PD groups were compared via parametric and non-parametric statistical tests. Then, the effects of group on frequency of falls, recurrent fall status, and likelihood of consequential falls, accounting for covariates (gender, age, and number of prescription medications) were investigated. Specifically, linear and logistic Generalized Estimating Equation (GEE) models were used, considering the dependence of longitudinal data as a cluster to correct confidence intervals. In our case, the individuals were the cluster. No missing data were observed.

*Timing of falls*: Time of day (TOD) & time of year across groups were first assessed via contingency tables and chi-square tests across groups. To further analyze differences in TOD, accounting for covariates, data were dichotomized into Day (morning, forenoon, or afternoon) and Night (evening or night). Then, a logistic GEE model was used to assess the impact of PD on TOD, accounting for age, gender, and number of medications. When covariates were observed to relate to TOD, additional contingency tables were created.

## Results

Participant characteristics, including fall frequencies are described across groups, and are shown in Table 1. Average age across groups was 83.7 years (range = [59.0–100.0]). Those with probable PD were significantly younger than controls (*p* = 0.012), but gender and number of medications prescribed were similar across groups (p’s > 0.1). The total number of recorded falls with and without consequence across PD and gender are shown in Table 2.

**Table 1:** Participant characteristics and fall counts.

	Level	Overall	Control	Probable PD	*p*
*N*		431	401	30	
Gender (Count (%))	Male	162 (37.7)	146 (36.5)	16 (53.3)	0.101
	Female	268 (62.3)	254 (63.5)	14 (46.7)	
Fall count (median [IQR])*		2.00 [1.00, 4.50]	2.00 [1.00, 4.00]	2.50 [1.25, 5.50]	0.289
Consequential fall count (median [IQR])*		1.00 [0.00, 1.00]	1.00 [0.00, 1.00]	1.00 [0.00, 2.00]	0.492
Recurrent faller status	No	1a58 (36.7)	150 (37.4)	8 (26.7)	0.327
	Yes	273 (63.3)	251 (62.6)	22 (73.3)	
Number of medications (mean (SD))		16.50 (6.85)	16.46 (6.92)	16.97 (6.03)	0.699
Age (mean (SD))		83.77 (7.86)	84.03 (7.85)	80.29 (7.34)	0.012
Observation period (mean (SD))		2.51 (0.79)	2.52 (0.78)	2.46 (0.89)	0.682

*non-parametric test.

**Table 2:** Fall counts within the 401 control and 30 probable PD participants.

	Control	Probable PD	*p*
*N* (total falls)	1644	154	
N (consequential falls (%))	433 (26.3)	38 (24.7)	0.724
Falls in Female Participants (%)	954 (58.0)	82 (53.2)	0.288

As shown in Tables 1 and 2, the number of falls (all falls and falls with consequence) and the rates of recurrent faller status were not different across groups (p’s > 0.28). GEE models further assessed the relationship between group and fall counts and recurrent status, accounting for age and number of medications. Regarding fall counts, PD group did not predict falls (*p* = 0.32). However, age and gender significantly predicted fall counts (*p* = 0.026 and *p* = 0.025, respectively), such that older adults and women fell more than younger or male counterparts. No variables were significant predictors of recurrent fall status (p’s > 0.118). Finally, the likelihood that falls resulted in a consequence was also not predicted by PD group or covariates (age, gender, or number of medications, p’s > 0.14). Full model outputs for number of falls, recurrent faller status, and consequential falls are shown in supplemental Tables 1, 2 and 3 respectively.

**Fig. 1:**
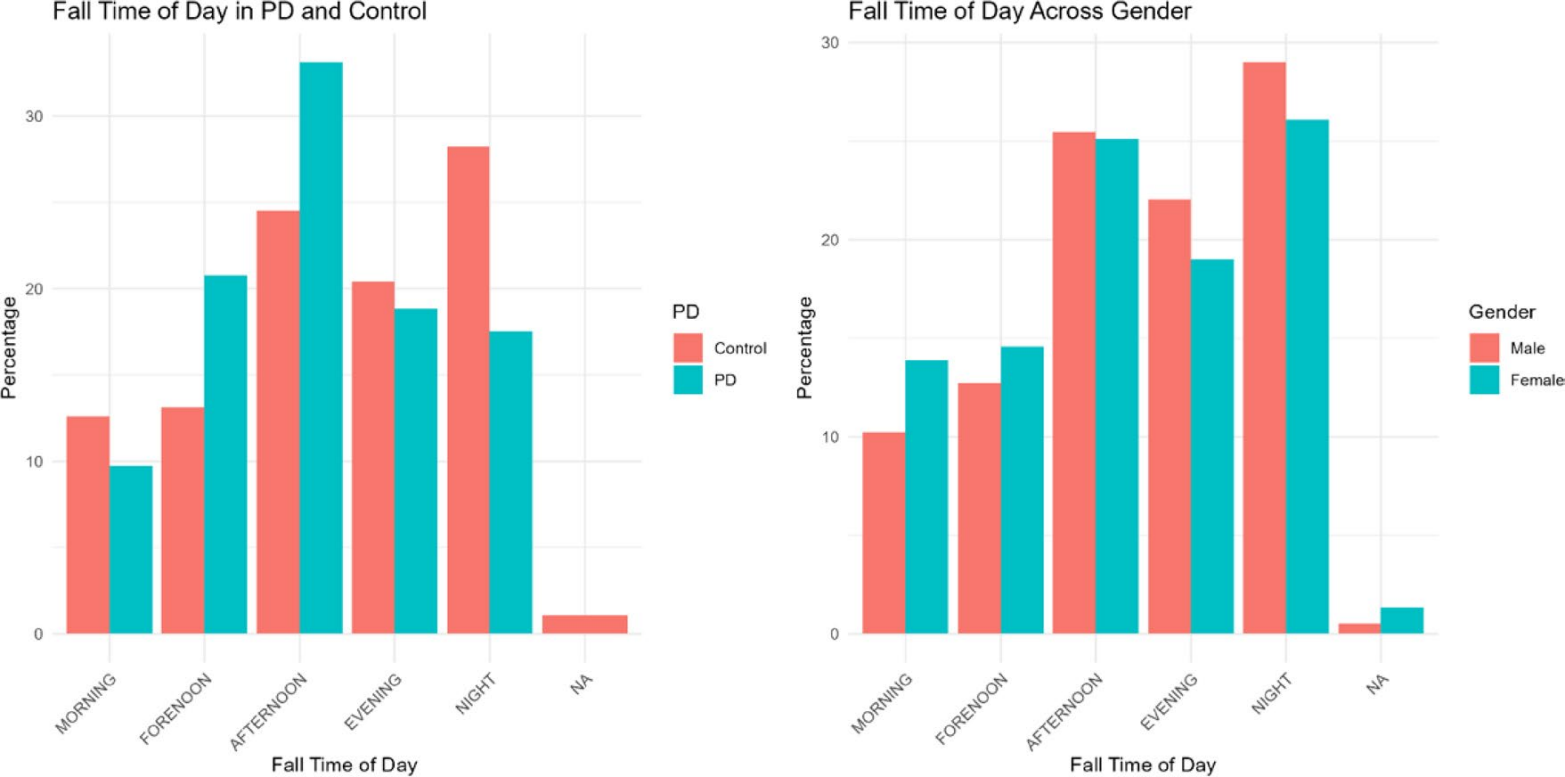
Fall time of day across those with and without probable PD (left) and gender (right). Chi-square tests indicate statistically significant effects of both PD and gender on frequency of falls throughout the day. Of note, morning, forenoon, and evening are 3 h windows, afternoon is a 6 h window, and night is an 8 h window.

Next, we assessed the effect of probable PD on fall time of day. First, contingency tables showed a statistically significant effect of both PD group (ᵪ^2^ = 16.9, *p* = 0.002) and, to a lesser extent, gender (ᵪ^2^ = 3.86, *p* = 0.049), on fall time of day, using the 5 categorizations (morning, forenoon, afternoon, evening, night). Specifically, those in the probable PD group were more likely to fall in the forenoon and afternoon and less likely to fall at night. Females were more likely to fall in the morning and forenoon, and less likely to fall in the evening and night (Fig. 1).

**Fig. 2:**
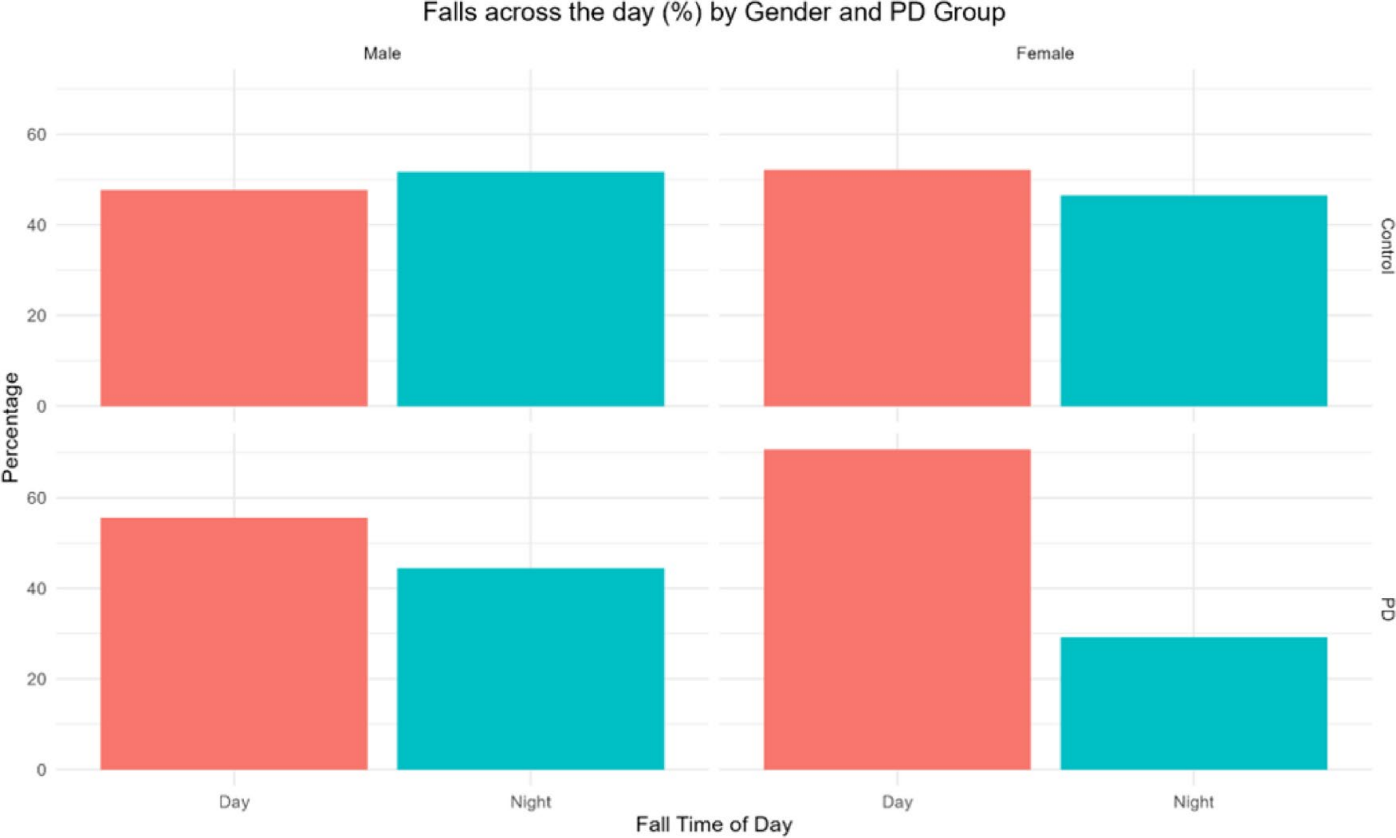
Dichotomized fall time of day (night- 18:00–6:00; day- 6:00–18:00), plotted across probable PD group and gender. Analyses suggest that people with Probable PD and females were more likely to experience a fall during the day than at night.

To further assess whether probable PD predicted fall TOD, accounting for gender, age, and medication, dichotomized fall TOD (day and night) data were used. Chi-squared analyses confirmed that dichotomized fall time of day was impacted by PD group and gender separately (ᵪ^2^ = 8.8, *p* = 0.003, and ᵪ^2^ = 5.3, *p* = 0.021, respectively, Supplemental Fig. 1). Then, a GEE model showed that probable PD group and gender (but not age or number of medications) were statistically significant predictors of dichotomized fall time of day. Specifically, people with probable PD were less likely to fall at night compared to day (*p* = 0.015, OR = 0.61). Similarly, female participants tended to fall less frequently at night than the day (*p* = 0.032, OR = 0.73; Fig. 2). Full GEE model outputs are reported in Supplemental Table 4.

**Fig. 3:**
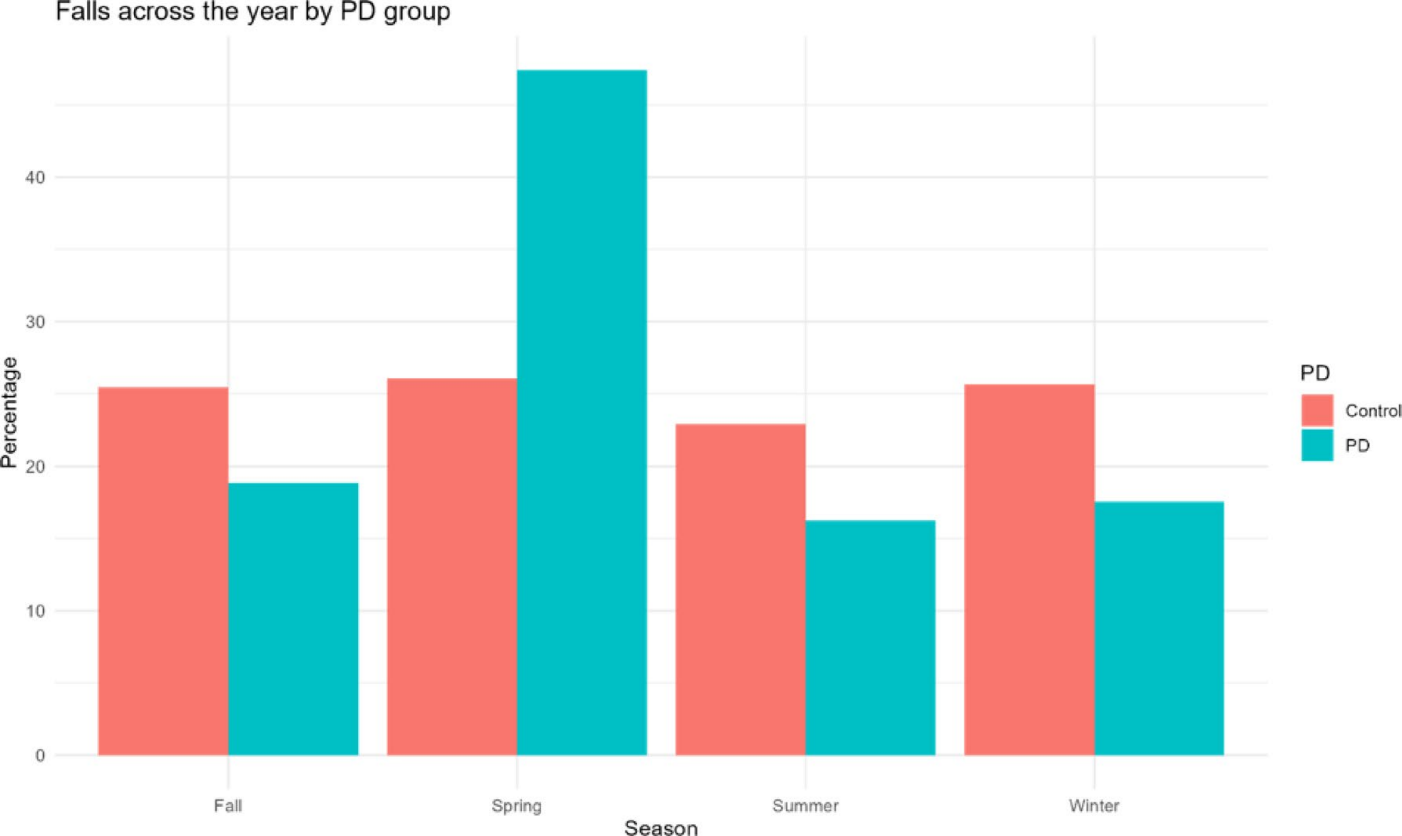
Percentage of falls in control and probable PD participants across seasons.

Finally, a chi-square test assessed time of year of falls across PD groups. People with probable PD had disproportionately more falls in the spring than other seasons, while those without PD had consistent falls throughout the year (ᵪ^2^ = 32.1, *p* < 0.001; Fig. 3). A follow-up contingency table for each month throughout the year showed similar results and is included in Supplemental Table 5.

## Discussion

Identifying the circumstances surrounding falls can be helpful to understand how and why falls occur. The current data suggest that over an approximately 2.5 year, prospective assessment of Swedish older adults who have fallen, people with probable PD fall more frequently during the daytime (and particularly during midday) than non-PD peers. Further, people with probable PD fell more frequently in the spring than other seasons, while season had little effect on falls of non-PD peers. This work is consistent with current literature, extending knowledge by 1) assessing an older (mean age, 83.7 years) population of fall-prone adults, 2) incorporating a relatively long follow-up period, 3) directly comparing the PD cohort to a large sample of people unlikely to have PD, and 4) showing that, in Sweden, PwPD may be more likely to fall in the spring than other seasons . Characterizing the timing (across the day and year) of falls in PwPD can assist with our understanding of why falls occur in these populations and could have clinical implications for prevention and care planning. For example, identification of time of day (or year) when falls are most common can facilitate times when individuals may need more assistance for safe and effective mobility.

We observed that individuals who were prescribed PD medications to fell more frequently during the daytime than the evening. In particular, PD participants fell more frequently in the afternoon which is consistent with both retrospective^8^ and prospective^4,9^ assessments of fall circumstances in PwPD. We extend this work in several ways. First, confirmation of previous findings in a relatively long follow-up period (mean 2.5 years) in a Swedish cohort underscores the robust nature of the finding. Further, we demonstrate the increased prevalence of daytime falls in those with probable PD was significantly larger than in non-PD peers. This is despite controls not falling more frequently and having been prescribed a similar number of medications (suggesting similar overall health^15^). Finally, previous reports focused on PwPD who were younger (mean ages of 60^6^ and 70^4^) with low to moderate fall risk. The current cohort were 80 (PD) and 84 (non-PD) years old and all participants had at least one registered fall. Extending previous results to older, more frail individuals is important to better understand causes of falls throughout the disease course. The rationale for the increased day-time fall-risk in people with PD is unclear. However it is possible that this relationship is due to an interaction between medication use, poor balance, and activity level. Fall risk increases with activity level^16^, and there is typically more activity during the day than nighttime. Further, PwPD often increase activity during ON medication times^17^, and PD medication can, in some patients, result in dyskinesias, increasing fall risk^18^. As such, the increased daytime activity and dyskinesias that can occur via PD medications could have contributed to daytime fall risk. Interestingly, we also observed a *smaller* proportion of falls in PD in the evening compared to adults. This finding is somewhat counterintuitive given the increase in OFF medication at night, difficulty with urinary frequency. Although the rationale for this finding is not fully understood, activity level may, again influence the relationship, such that PwPD may be moving less in the evening than their control counterparts, thus ultimately reducing the likelihood of falls, However, this hypothesis is speculative, and requires additional, specific testing. In particular, assessment of physical activity across groups and time would assist in removing the confound of activity to better understand other fall-risk behaviors.

We observed people unlikely to have PD to exhibit similar fall frequently across day and night, , . This is partially inconsistent with previous work, which suggests falls are more frequent during the day in this group^19,20^. This may be due to the age of participants, as the typical age of those studies previously was 60–80, while the mean age in the current cohort was 84. Indeed, increased age is related to elevated time spent indoors^21^, and indoor falls are associated with more night-time falls^4,6^. We also observed a subtle but statistically significant effect of gender on fall time of day, such that females fell more during the day than males. Previous work has not reported a statistically significant effect of gender on fall time of day, although Aoyagi and colleagues did observe a small, non-significant increase in the same direction as the current results^19^. Previous reports are indirectly conflicting with this finding, as they indicate that males more frequently fall outside, and outside falls are more common during the day ^4^. However as noted above, the age of participants may have impacted these findings, as the current, older cohort likely spent less time outside regardless of gender^22^.

Older adults with probable PD living in Sweden exhibited a distinct increase in falls during the spring, while non-PD peers had similar fall rates throughout the year. To our knowledge, fall rates across the year have not been previously reported in PwPD, and the rationale for these findings are unknown. Previous work has suggested that PD symptoms (such as the UPDRS part III), mobility, and quality of life were all worse in the spring compared to other seasons^23^. It is possible that season-specific worsening of PD symptoms, paired with the increased activity typical of springtime more generally could have resulted in increased falls in the PD group compared to non-PD controls, who may have had less season-specific risk. However, additional work will be necessary to confirm or contradict this relationship and proposed rationale.

Finally, it was observed that the current cohort of probable PD did not fall more frequently than non-PD peers. Further, while a larger percentage of PD were recurrent fallers than controls (73% vs. 63%, respectively), this difference was not significant. Previous work suggests that while retrospective fall history can be similar in PD and control populations, prospective fall counts and the number of recurrent fallers are typically higher in PwPD^1^. The lack of influence of PD on number of falls and frequency of recurrent fallers in the current study may be related to the characteristics of the control cohort. First, all participants (both PD and controls) included in the study had at least 1 registered fall, resulting in a group with higher fall-risk than the typical older adult population. Further, the control subjects were, on average, 4 years older than the PD group and as noted above, both groups were older than previously reported cohorts. Finally, although specific co-morbidities were not captured, the total number of unique medications prescribed over the approximately 2.5-year observation period was high (16 on average), and similar across groups. This metric has been shown to track with overall heath^15^, and suggests that both groups were likely in declining health. This is consistent with previous work indicating that the Senior Alert cohort, from which the current data are pulled, are relatively frail^13^. These possible explanations aside, the lack of difference in fall frequency across groups may indicate that this sample of PD participants is not representative of PwPD within Sweden or elsewhere. As such, previously reported PD-control comparisons should be treated with caution.

Several strengths and limitations of the current study should be mentioned. Regarding strengths, the current analysis utilized a relatively long (~ 2.5 year), prospective dataset, with 431 older adults included in the analysis. Over this period, 1798 falls were reported. Further, the individuals included were relatively old (mean age = 83.7 years), and all individuals had experienced at least 1 fall over the observation period. This cohort is important to study, as they are at particularly high fall risk. In addition, the sample was drawn from a nationally representative cohort of Swedish older adults, not limited to a single city or region. Regarding limitations, our sample contained a relatively small number of probable PD participants. Further, we were not able to contact these individuals to confirm their PD diagnosis. Although somewhat offset by the long, prospective follow-up period (154 falls were registered in the PD cohort alone), and stringent PD categorization (only people taking dopamine or dopamine derivatives were included in the probable PD group), this remains an important weakness. Second, falls were registered by healthcare staff rather than patients or caregivers. Therefore, falls may have been under-estimated. However, self-reporting of falls (even prospective) can be problematic and falls are often underreported, and external fall-reporting carries some benefits. In particular, falls were registered by healthcare staff at multiple types of healthcare facilities (i.e., not just hospitals), increasing the scope of the database. Although the reporting accuracy across these facilities may vary^11^, it provides many venues in which falls could be identified. Third, as noted above, our PD cohort did not fall more than older adults, perhaps because the higher age of the control group. While all analyses included age as a co-variate, the lack of an impact of PD on fall frequency suggests that this cohort may not be generalizable to other PD cohorts. Fourth, physical activity levels were not able to be captured within or across days. Physical activity impacts fall frequency, and should be collected and included in analytic models for future studies. Finally, co-morbidities were not available to include as co-variates in the analysis. Number of medications (a surrogate to overall health), was however included as a covariate, somewhat reducing the impact of this limitation.

## Conclusion

We observed that among Swedish older adults who have fallen, people prescribed PD medications fell more frequently during the day than night and during the spring than other seasons. This information can be used in conjunction with other fall characterization work to better predict and prevent falls in this population ^24^.

## Supplementary Information

Below is the link to the electronic supplementary material.

Supplementary Material 1

## Data Availability

Data are from a Swedish registry. As such, subject level data cannot be made available. However, group-level data will be shared via reasonable request via the corresponding author- Daniel Peterson.
