# Supplementary material for: Characterizing the time of day and year of falls in people with probable Parkinson’s disease
Source: Sci Rep. 2025 Sep 12;15:32508. doi: 10.1038/s41598-025-17752-1 (PMC12432150; doi:10.1038/s41598-025-17752-1)
Supplement: Supplementary file 1 — Supplementary Material 1 [file 41598_2025_17752_MOESM1_ESM.docx]

**Supplemental Table 1**: Full linear model output to assess the effects of PD group, gender, age, and number of medications on total fall counts.

Call: gee(formula = counts ~ pdgrp + gender + ageatstart + num_medications,

data = fallsandmeds, cond = FALSE, clusterid = "subid")

Model: counts ~ pdgrpPD + genderFemale + ageatstart + num_medications

Link function: identity

Estimate Std. Error z value Pr(>|z|)

(Intercept) -2.00978 3.34184 -0.601 0.5476

pdgrpPD 1.21348 1.22839 0.988 0.3232

genderFemale -1.15967 0.51755 -2.241 0.0250 *

ageatstart 0.08278 0.03734 2.217 0.0266 *

num_medications -0.01035 0.03411 -0.303 0.7617

---

Signif. codes: 0 ‘***’ 0.001 ‘**’ 0.01 ‘*’ 0.05 ‘.’ 0.1 ‘ ’ 1

430 complete observations used

**Supplemental Table 2**: Full linear model output to assess the effects of PD group, gender, age, and number of medications on recurrent faller status.

Call: gee(formula = rec_faller ~ pdgrp + gender + ageatstart + num_medications,

link = "logit", data = fallsandmeds, cond = FALSE, clusterid = "subid")

Model: rec_fallerYes ~ pdgrpPD + genderFemale + ageatstart + num_medications

Link function: logit

Estimate Std. Error z value Pr(>|z|)

(Intercept) -0.964532 1.195865 -0.807 0.420

pdgrpPD 0.529685 0.424131 1.249 0.212

genderFemale -0.300078 0.222530 -1.348 0.178

ageatstart 0.021557 0.013800 1.562 0.118

num_medications -0.007876 0.015214 -0.518 0.605

430 complete observations used

Odds Ratio 2.5 % 97.5 %

(Intercept) 0.3811616 0.03657522 3.972203

pdgrpPD 1.6983969 0.73963663 3.899958

genderFemale 0.7407607 0.47891689 1.145766

ageatstart 1.0217910 0.99452365 1.049806

num_medications 0.9921546 0.96300696 1.022185

**Supplemental Table 3**. Full linear model output to assess the effects of PD group, gender, age, and number of medications on consequential faller status

Call: gee(formula = FALLCONSEQUENCES ~ pdgrp + gender + ageatstart +

num_medications, link = "logit", data = allFallsData, cond = FALSE,

clusterid = "subid")

Model: FALLCONSEQUENCESYes ~ pdgrpPD + genderFemale + ageatstart + num_medications

Link function: logit

Estimate Std. Error z value Pr(>|z|)

(Intercept) -1.020351 1.014874 -1.005 0.315

pdgrpPD -0.083288 0.230413 -0.361 0.718

genderFemale 0.231286 0.158306 1.461 0.144

ageatstart -0.003718 0.011625 -0.320 0.749

num_medications 0.010337 0.011192 0.924 0.356

1798 complete observations used

Odds Ratio 2.5 % 97.5 %

(Intercept) 0.3604685 0.04931799 2.634689

pdgrpPD 0.9200861 0.58573396 1.445295

genderFemale 1.2602191 0.92405146 1.718684

ageatstart 0.9962887 0.97384514 1.019250

num_medications 1.0103906 0.98846800 1.032799

**Supplemental Figure 1**: Dichotomized time of day data for PD and gender groups. Chi-squared analyses for the PD and gender were statistically significant (x2 = 8.8, p = 0.003, and x2 = 5.3, p = 0.021, respectively).


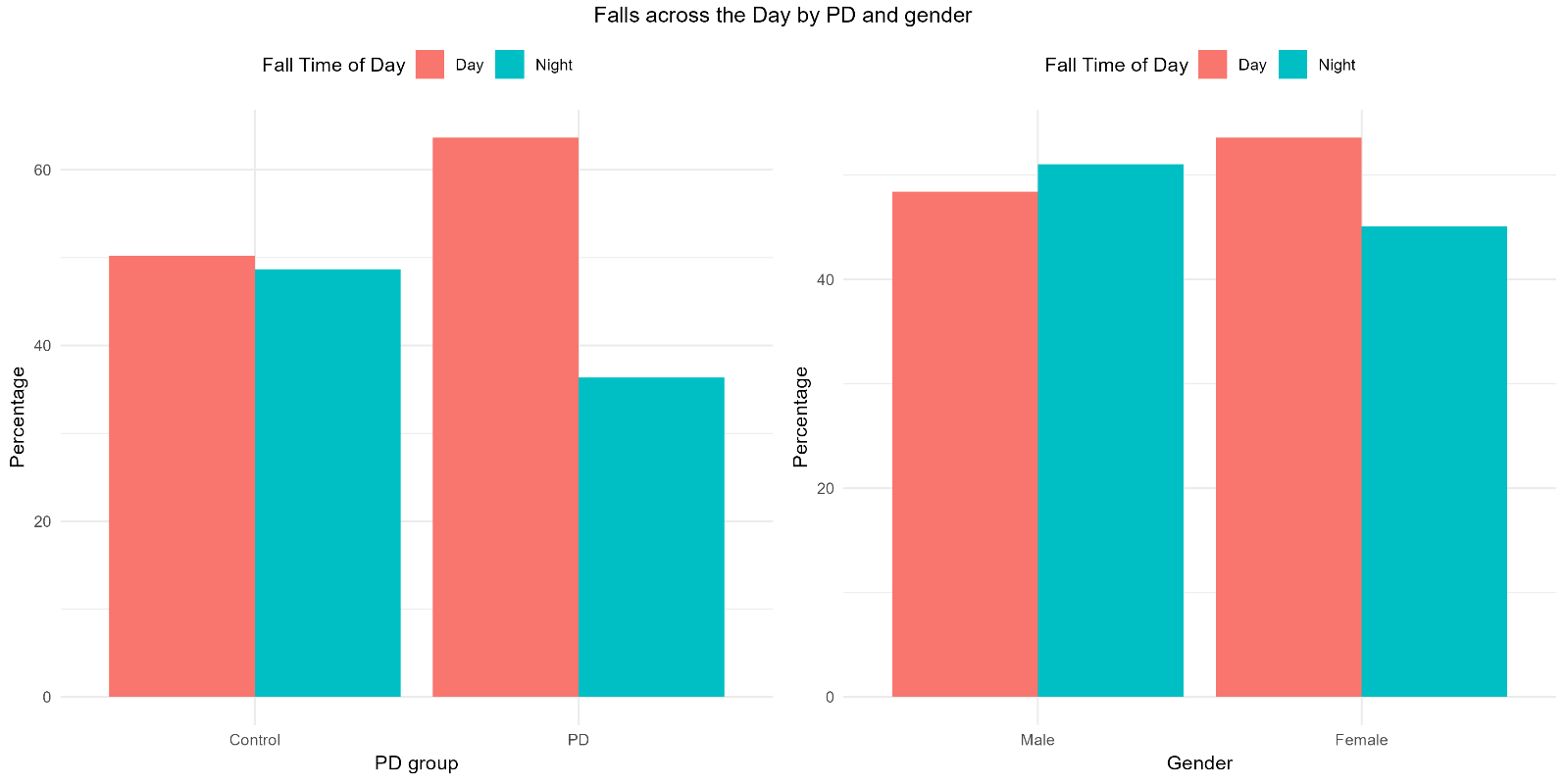


**Supplemental Table 4**: Full model outputs showing the effect of PD group, gender, age, and number of medications on fall time of day, dichotomized to day or night.

Estimate Std. Error z value Pr(>|z|)

(Intercept) -1.169234 0.746996 -1.565 0.1175

pdgrpPD -0.500724 0.204954 -2.443 0.0146 *

genderFemale -0.308677 0.143633 -2.149 0.0316 *

ageatstart 0.013043 0.008689 1.501 0.1333

num_medications 0.012700 0.009071 1.400 0.1615

---

Signif. codes: 0 ‘***’ 0.001 ‘**’ 0.01 ‘*’ 0.05 ‘.’ 0.1 ‘ ’ 1

1780 complete observations used

Odds Ratio 2.5 % 97.5 %

(Intercept) 0.3106048 0.07183959 1.3429270

pdgrpPD 0.6060918 0.40558453 0.9057230

genderFemale 0.7344178 0.55422016 0.9732045

ageatstart 1.0131281 0.99602115 1.0305288

num_medications 1.0127813 0.99493521 1.0309474

**Supplemental Table 5**: Contingency table showing falls in probable and non-probable PD across each month.

Stratified by pdgrp

level Control PD p test

n 1644 154

month (%) 1 135 ( 8.2) 5 ( 3.2) <0.001

2 129 ( 7.8) 15 ( 9.7)

3 158 ( 9.6) 34 (22.1)

4 146 ( 8.9) 22 (14.3)

5 124 ( 7.5) 17 (11.0)

6 133 ( 8.1) 7 ( 4.5)

7 128 ( 7.8) 10 ( 6.5)

8 115 ( 7.0) 8 ( 5.2)

9 166 (10.1) 11 ( 7.1)

10 141 ( 8.6) 8 ( 5.2)

11 111 ( 6.8) 10 ( 6.5)

12 158 ( 9.6) 7 ( 4.5)
